# A Thorough Synthesis of Phage Therapy Unit Activity in Poland—Its History, Milestones and International Recognition

**DOI:** 10.3390/v14061170

**Published:** 2022-05-28

**Authors:** Maciej Żaczek, Andrzej Górski, Beata Weber-Dąbrowska, Sławomir Letkiewicz, Wojciech Fortuna, Paweł Rogóż, Edyta Pasternak, Ryszard Międzybrodzki

**Affiliations:** 1 Bacteriophage Laboratory, Department of Phage Therapy, Ludwik Hirszfeld Institute of Immunology and Experimental Therapy, Polish Academy of Sciences, 53-114 Wrocław, Poland; andrzej.gorski@hirszfeld.pl (A.G.); beata.weber-dabrowska@hirszfeld.pl (B.W.-D.); edyta.pasternak@hirszfeld.pl (E.P.); ryszard.miedzybrodzki@hirszfeld.pl (R.M.); 2 Phage Therapy Unit, Ludwik Hirszfeld Institute of Immunology and Experimental Therapy, Polish Academy of Sciences, 53-114 Wrocław, Poland; letkiewicz1@o2.pl (S.L.); wojciech.fortuna@hirszfeld.pl (W.F.); pawel.rogoz1@gmail.com (P.R.); 3 Infant Jesus Teaching Hospital, Medical University of Warsaw, 02-005 Warsaw, Poland; 4 Department of Health Sciences, Jan Długosz University in Częstochowa, 42-200 Częstochowa, Poland; 5 Department of Neurosurgery, Wrocław Medical University, 50-556 Wrocław, Poland; 6 Bioethics Committee, Ludwik Hirszfeld Institute of Immunology and Experimental Therapy, Polish Academy of Sciences, 53-114 Wrocław, Poland; 7 Department of Clinical Immunology, Medical University of Warsaw, 02-006 Warsaw, Poland

**Keywords:** phage therapy, phage research, experimental treatment, Hirszfeld Institute, Phage Therapy Unit, Polish history, bioethics committee, compassionate use

## Abstract

The year 2020 marked 15 years of the Phage Therapy Unit in Poland, the inception of which took place just one year after Poland’s accession to the European Union (2004). At first sight, it is hard to find any connection between these two events, but in fact joining the European Union entailed the need to adapt the regulatory provisions concerning experimental treatment in humans to those that were in force in the European Union. These changes were a solid foundation for the first phage therapy center in the European Union to start its activity. As the number of centers conducting phage therapy in Europe and in the world constantly and rapidly grows, we want to grasp the opportunity to take a closer look at the over 15-year operation of our site by analyzing its origins, legal aspects at the local and international levels and the impressive number and diversity of cases that have been investigated and treated during this time. This article is a continuation of our work published in 2020 summarizing a 100-year history of the development of phage research in Poland.

## 1. Preface

Phage therapy in Poland had been conducted long before the initiation of the Phage Therapy Unit (PTU) and is inevitably associated with the Hirszfeld Institute, an internationally recognized research center founded in 1952 in Wrocław, Poland [1]. Undeniably, the postwar activity of phage researchers at the Hirszfeld Institute constituted a prerequisite for establishing the Phage Therapy Unit, the first ethically approved phage treatment facility in Europe, in the form we know today. The Hirszfeld Institute remains a home for the Phage Therapy Unit to this day as well as for the newly created Phage Therapy Department that includes two phage laboratories.

Analysis of the activity of the PTU in Poland requires shedding more light on applicable legal and ethical aspects of experimental treatment and conditions applicable after Poland’s accession to the European Union. Prior to that event, phage treatment was not considered to be a therapy under specific legal conditions. For nearly 50 years, patients seeking help reported to the Hirszfeld Institute and, based on their condition and phage typing results, personalized phage lysates were given to them in good faith. A similar approach was used in microbiology laboratories for the production of individual autovaccines [2,3]. Moreover, phage preparations from the Wrocław center were supplied to hospitals across Poland [1,4]. This process was based on the long-term experience of phage researchers and the fact that phage therapy was considered safe. In 1987, Prof. Stefan Ślopek, M.D., Ph.D., the second Hirszfeld Institute director who greatly expanded phage research in Poland, reported that among 550 analyzed cases in the years 1981–1986 at the Hirszfeld Institute, adverse events were “rarely encountered” [5]. Overall, more than 1000 patients received phage treatment in the years 1954–1987 with a reported success rate of 84–97% [6,7,8,9,10,11,12,13]. Although such an approach was widely acceptable in those days, it would not last under today’s rules of evidence-based medicine. The rapidly changing perspective, inevitably associated with progress in medical sciences and the increased number of medical experiments involving human subjects, forced changes to the treatment regimen applied outside standard practice [4].

It should be noted that the above-mentioned indulgent approach does not mean that phage therapy before the PTU’s establishment was conducted completely out of any medical control. From a letter dated 5 April 1986, addressed to Prof. Stefan Ślopek, the then director of the Hirszfeld Institute, we learn about the District Committee for Monitoring of Human Trials that issued a favorable decision regarding basic medical research involving humans. However, we can easily assume that the legal and medical landscape for phage treatment has changed significantly over the past 35 years [4]. Without a doubt, the initiation of the PTU introduced higher ethical and legal standards, which will be described in more detail in this article.

## 2. Methodology

Our research is mainly based on the documentation collected in the Bacteriophage Laboratory regarding the phage-related assays (summaries on phage typing, phage collection and phage lysates provided to patients) performed on a daily basis. The second source of documentation was the medical history of patients registered at the PTU. The third source in the form of internal historical documents and publicly available articles shed more light on PTU initiation and changes made to the treatment regimen after Poland’s accession to the EU (bioethics committee letters, protocols, informed consent forms). Finally, the experience of the Bacteriophage Laboratory and PTU staff, in particular of Prof. Andrzej Górski, M.D., Ph.D. and Assoc. Prof. Ryszard Międzybrodzki, M.D., Ph.D, provided a more detailed insight into the practical side of phage therapy conducted at the PTU, its legal basis and key aspects. Human resource documents available at the Hirszfeld Institute were, in turn, the source of data regarding team members, their achievements and their employment status.

## 3. Legal Aspects of Experimental Treatments

According to Aulisio [14], three cases that took place in the second half of the 20th century in the USA—the God Committee, and the Quinlan and Cruzan cases—served as prerequisites for bioethics committees (BCs). In the early 1980s, only 1 percent of US hospitals had operating BCs, a number that skyrocketed to over 90 percent within the next 20 years. The first above-mentioned case from 1962 concerned selection criteria for dialysis patients whose number was greater than the capacity of the dialysis unit. Following the large-scale consultations, the people who adopted the criteria were soon named “The God Committee”, which clearly emphasized the importance and significance of the decisions they had to make. The Quinlan case from 1976 and the Cruzan case from 1990 dealt with equally difficult verdicts on when it is the right time to unplug life-sustaining devices from an unconscious patient. Obviously, these cases were only a modest representation of many similar ones that clinicians had to deal with on a daily basis, and their number was growing rapidly. BCs, consisting of a panel of multidisciplinary experts, seemed to be the best way to assist with such difficult decisions concerning ethical issues. Currently, BCs are a permanent element of the clinical landscape, being present in more than 95% of hospitals [15]. The work of BCs has gained wide acceptance among healthcare professionals, and even with some concerns raised by physicians regarding the level of expertise of some BC members, these days, it is difficult to imagine modern medicine without these institutions [16].

On 4 April 2001, as part of a continuous process to develop principles in response to an increasing number of clinical trials, Directive 2001/20/EC of the European Parliament and of the Council was passed establishing laws, regulations and administrative provisions of the member states relating to the implementation of good clinical practice in the conduct of clinical trials on medicinal products for human use. Together with the subsequent Directive 2005/28/EC on Good Clinical Practice, it fundamentally reorganized the principles of conducting clinical trials in Europe [17]. However, in parallel with the extensive growth of the clinical research industry in Europe and outside of the continent, there was an unmet legislative need for so-called compassionate treatment based on the use of unapproved drugs with a potential beneficial effect for the patient [18]. The term “unapproved drug” denotes a drug with unproven safety and efficacy which nowadays seems to be a sufficient reason for the treatment to be closely monitored and regulated by law. The problem was even more noticeable in terms of phage therapy utilizing virus particles that are quite different from what we tend to call medicinal products [19]. Existing documents did not create a legal framework for such a type of treatment, and each country was supposed to set their own rules and procedures. Compassionate treatment under different names is also available outside Europe, in the USA under the name of expanded access, in Canada through Special Access Programs and in Australia in the form of the Authorized Prescriber Scheme and the Special Access Scheme [20]. Notably, the recent pandemic outbreak of COVID-19 demonstrated the importance of compassionate use treatment in emerging situations. While pharma companies were desperately seeking a vaccine, and no one could possibly know when it would be available to the public, patients died on a daily basis as a result of a lack of appropriate treatment. It is worth mentioning that once the COVID-19 vaccines were developed, an initial mass-scale vaccination took place in the form of conditional approval granted by the regulatory authorities [21]. In other words, healthcare systems in several countries around the globe faced the situation of a not fully approved medicinal product being administered to millions of people. Hence, experimental treatment seems to be more relevant these days than one could have possibly predicted. The phage model of compassionate use has already paved the way for the first clinical trials conducted at an increasing number of research centers and is closely associated with the Phage Therapy Unit activity in Poland. The treatment model employed in Poland has already been proposed as the potential solution to meet the growing medical need in the USA [22]. The importance of compassionate treatment was also noted by the European Medicine Agency, which on 19 July 2007 issued a Guideline on Compassionate Use of Medicinal Products, pursuant to Article 83 of Regulation (EC) No 726/2004. The main purpose of this guidance was to facilitate the availability of new treatment options under development to patients. Such compassionate programs should be governed by individual member states’ legislation, and this is exactly how the PTU’s activity has been established in Poland.

## 4. Phage Therapy Unit

### 4.1. Establishment of PTU in Poland

According to Polish law, phage therapy is considered an experimental treatment and is based, in addition to Polish regulations, on the Declaration of Helsinki. This statement of ethical principles for medical research involving human subjects was adopted in 1964 by the 18th World Medical Association General Assembly and has been modified several times over the years to adapt to new conditions. It has become the basic set of principles for physicians who want to conduct medical research involving human subjects [23]. It also constitutes important ethical guidance about the use of drugs that have not yet obtained marketing authorization, including phage treatment [4,20]. An article of particular importance is article 37 entitled “Unproven Interventions in Clinical Practice”, which includes wording strictly referring to compassionate forms of treatment: “(...) *where proven interventions do not exist or other known interventions have been ineffective, the physician, after seeking expert advice, with informed consent from the patient or a legally authorised representative, may use an unproven intervention if in the physician’s judgement it offers hope of saving life, re-establishing health or alleviating suffering*.” [24] The Declaration of Helsinki has become a model for regulatory documents in different countries determining guidelines for experimental treatment. In Poland, such rules have been implemented in the Medical and Dental Professions Act of 5 December 1996, in particular Chapter 4 concerning medical experiments. In fact, the two above-mentioned documents, the Declaration of Helsinki and the Medical and Dental Professions Act, have constituted the basis for conducting treatment at the PTU since 2005 [4]. Additional documents include the Constitution of Poland and the ethical code of the Polish Medical Association.

**Figure 1:**
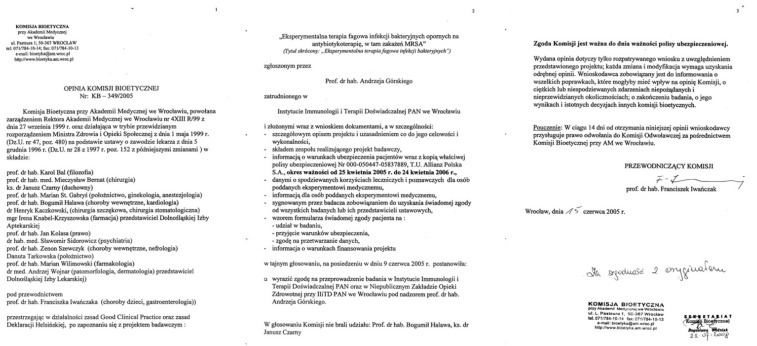
A decision dated 15 June 2005, authorizing the beginning of experimental phage treatment (officially named “Experimental phage therapy of drug-resistant bacterial infections, including MRSA infections”) at the PTU in Wrocław, Poland (in Polish).

The first major milestone that underlies the launch of the PTU in Wrocław, Poland, was an approval granted by the BC of the Medical Academy in Wrocław (today’s Wrocław Medical University). The decision of the BC No. 349/2005 dated 15 June 2005 (Figure 1) granted authorization for the project submitted by Prof. Andrzej Górski entitled “*Experimental phage therapy of drug-resistant bacterial infections, including MRSA infection*”, in adherence to the principles of Good Clinical Practice and the Declaration of Helsinki. It became the first ethically approved document regarding experimental phage treatment in the European Union. BC approval outlined every aspect of modern conduct of experimental treatment, including the composition and qualifications of the medical staff participating in the project, details of the policy of mandatory civil liability insurance, the patient information sheet and the informed consent form to participate in the medical investigation. Briefly, a medical doctor in Poland is allowed to apply phage therapy where proven therapeutic methods do not exist or have been ineffective (e.g., in MDR infections), provided that the patient or their legal representative has given informed consent. Since that important milestone, phage therapy has been conducted on an outpatient basis under the supervision of Prof. Andrzej Górski. The changes compared to phage treatment conducted before the PTU’s establishment included the direct care of patients who were required to attend follow-up visits associated with blood sample collection and clinical and laboratory monitoring. Although, due to a lack of funding, no clinical trial has been conducted at the PTU thus far, we were able to gather unique data that provide a solid basis for starting such clinical trials. This statement is in line with an opinion delivered by a former FDA commissioner emphasizing that, in parallel to clinical studies, the crucial and complementary role of high-quality observational studies should not be neglected [25]. Because pharma companies are cautious about investing in phage treatment, non-commercial clinical trials could be the solution. Unfortunately, in Poland, non-commercial clinical trials account for a negligible percentage of all clinical trials (one of the lowest numbers in the European Union). The main barrier to conducting non-commercial clinical trials in Poland is the lack of an efficient financing mechanism for this type of project, while the stringent requirements are the same for both commercial and non-commercial trials [26]. The appointment of the Medical Research Agency in 2019 brings hope for the long-awaited changes in this matter.

As mentioned above, the PTU’s core activity is based on Polish legislation, in particular the Medical and Dental Professions Act of 5 December 1996 which defines the rules and conditions for practicing the professions of a physician and dentist but also protects the patient’s rights, establishes the legal framework for conducting a medical experiment and characterizes the role of bioethics committees. In addition to the above-mentioned document, conducting medical experiments in Poland is based on: the Constitution of the Republic of Poland, the Criminal Code, the Pharmaceutical Law Act of 6 October 2001, the Regulation of the Minister of Health of 11 March 2005 on the detailed requirements of Good Clinical Practice, the Medical Code of Ethics and the Regulation of the Ministry of Health and Social Welfare of 11 May 1999 concerning the detailed rules of appointment, funding and mode of operation of BCs.

It must be emphasized that in the early 2000s, the regulatory landscape for phages significantly differed when compared to current efforts made by the phage community. Regular conferences (also in an online mode) where regulatory, commercial and scientific aspects of phage therapy are discussed have never been practiced before on such a large scale, mostly due to the limited interest in this type of treatment. This was recently described in detail by Verbeken and Pirnay [19]. The situation in Poland where contract research organizations (CRO) were just starting their activities was even worse [26]. Although the first BC in Poland was established at the Medical Academy in Gdańsk in 1979 as the Team for the Deontological Assessment of Scientific Research, the implemented provisions appointing BCs at medical academies, research units and district medical chambers came into force 20 years later in the form of the above-mentioned Regulation of the Ministry of Health and Social Welfare of 11 May 1999 [27]. Hence, one could confidently say that the opening of the PTU was an unprecedented move, both at a local and a global level. Although the legal situation in Poland regarding medical experiments is complex (regulations are included in different law acts and depend on the type of investigation), the Medical and Dental Professions Act remains the main legislative act defining the role of BCs [28]. As of 2020, there were 69 BCs in Poland, which shows the progress made in the field of experimental treatment in our country [29].

### 4.2. Organizational Structure, Team Members and Educational Activity

The PTU is part of the Medical Center, the unit subordinated to the Hirszfeld Institute of Immunology and Experimental Therapy, Polish Academy of Sciences (shortened to the Hirszfeld Institute) in Wrocław, Poland, which was created in parallel to the PTU’s establishment in 2005. The role of the Medical Center is to carry out outpatient health services (including cooperation with other medical entities) related to research conducted at the Hirszfeld Institute and using the results of basic research to develop new methods of prevention as well as diagnostic methods and new treatment strategies. Additionally, the units of the center take part in the postgraduate education of people practicing the medical profession. In the years 2014–2020, there were also two PTU branches in the southern region of Poland, in Częstochowa and Kraków, which were closed due to the COVID-19 pandemic outbreak and decreased patient interest. Currently, the Medical Center consists of three organizational units: the PTU, the Laboratory of Tissue Immunology (LTI) and the so-called COVID laboratory. While this paper is solely dedicated to the activity of the PTU, it is worth mentioning that since 1990, the LTI has been commercially testing patients for genetic predisposition to autoimmune diseases (including rheumatic diseases, uveitis, recurrent iritis, psoriatic arthritis, type 1 psoriasis, celiac disease) and received accreditation from the European Federation for Immunogenetics in 2018. In May 2020, the SARS-CoV-2 Infection Diagnostics Laboratory was initiated as a result of the COVID-19 pandemic outbreak. More information about the activity and organizational structure of the Medical Center of the Hirszfeld Institute in English is available online [30].

**Figure 2:**
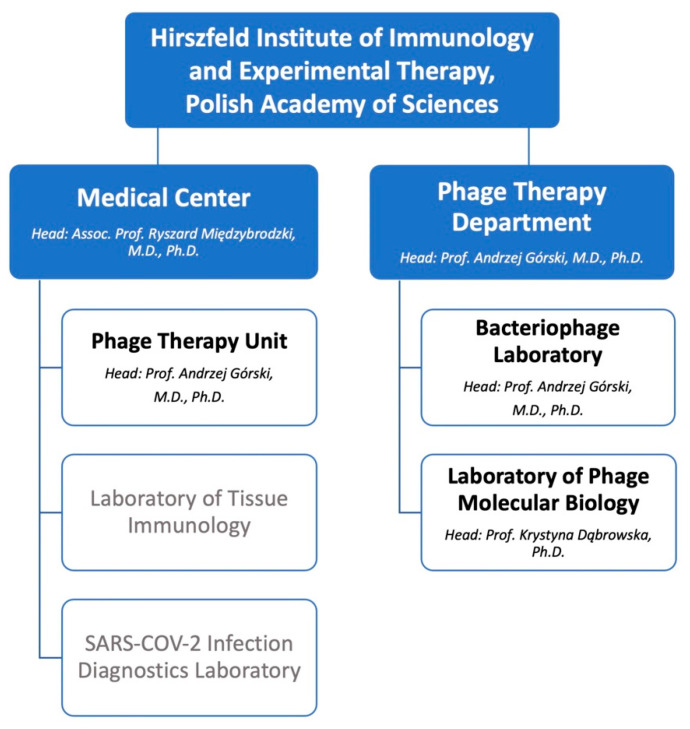
Organizational structure of phage-related units (highlighted in bold) within the Hirszfeld Institute as of April 2022 (along with the heads of individual units).

Teamwork has always been an essential capability for successful progress, and the PTU is not an exception. From the first day of its operation, the PTU has employed several physicians specialized in endocrinology, general surgery, gynecology, pediatrics, orthopedics, medical microbiology and urology. As of April 2022, there were five physicians, including Prof. Andrzej Górski, M.D., Ph.D., internal medicine and clinical immunology specialist and head of the Phage Therapy Unit, and Assoc. Prof. Ryszard Międzybrodzki, M.D., Ph.D., head of the aforementioned Medical Center of the Hirszfeld Institute. In addition, the PTU team consists of a medical secretary responsible for patient registration (before 2017, there was one secretary who shared responsibilities between the PTU and the Bacteriophage Laboratory) and a coordinator for phage preparations on behalf of the Bacteriophage Laboratory, Beata Weber-Dąbrowska, Ph.D. The work of the PTU is closely related to and sometimes supplemented by two phage laboratories—the Bacteriophage Laboratory and the Laboratory of Phage Molecular Biology—included in the newly created Phage Therapy Department which is led by Prof. Andrzej Górski. Details of the organizational structure of phage-related units within the Hirszfeld Institute are presented in Figure 2.

As of April 2022, the member composition of the Phage Therapy Department consisted of 12 research workers specialized in medical and life sciences (2 full professors, 1 associate professor, 5 Doctors of Philosophy, 4 Masters of Science), 1 secretary, 1 technician and 5 Ph.D. students. These numbers do not include numerous M.Sc. and Ph.D. students and interns as well as two Fulbright Scholar Program participants who were involved in research activity in the past. Moreover, the work of the Phage Therapy Department is continuously strengthened by employees participating in research programs funded by a vast range of financing institutions. Due to the nature of research grants, the number of such employees varies over time. Despite the periodic nature of such contracts, these numbers cannot be underestimated. Between 2005 and 2021 the Bacteriophage Laboratory was visited by 102 students and researchers as part of internships and scholarships. The majority of them studied at Wrocław universities (mainly the University of Wrocław, Wrocław University of Science and Technology and Wrocław University of Environmental and Life Sciences), but some were also international students from Spain, the United Kingdom and the United States. Furthermore, during this time, 32 students defended their M.Sc. theses, the majority of which were focused on phage molecular biology under the supervision of Prof. Krystyna Dąbrowska, and 6 students defended their Ph.D. projects in the immunology (supervised by Prof. Andrzej Górski) and molecular biology (supervised by Prof. Krystyna Dąbrowska) fields. Currently, seven students are pursuing their Ph.D. projects in both laboratories included in the Phage Therapy Department supervised by Prof. Andrzej Górski, Prof. Krystyna Dąbrowska and Assoc. Prof. Ryszard Międzybrodzki.

### 4.3. Treatment Regimen

As previously pointed out, treatment of patients seeking help at the Hirszfeld Institute has dramatically changed since the initiation of the PTU in 2005. In accordance with BC approval, patients are provided with an information sheet and informed consent form before any medical procedures are implemented, in a similar way to how patients are enrolled in clinical trials. The protocol specifies details concerning visits and follow-up visits as well as conditions for starting and terminating patients’ participation in the treatment. One of the differences between a clinical trial and phage therapy conducted at the PTU is the cost patients need to cover in order to receive phage preparations. Additionally, patients are not reimbursed for their in-person presence at the PTU. One could imagine that this significantly affects the number of visits, especially those in the follow-up period and in the case of patients from abroad. Nevertheless, physicians make efforts to make these visits relatively regular, which is reflected in our publications summarizing the therapeutic outcomes [25,31] including a plethora of articles on humoral response to phage preparations describing phage neutralization and the formation of antiphage antibodies before, during and after phage treatment [32,33,34,35,36,37].

Developed by Assoc. Prof. Ryszard Międzybrodzki and approved by Prof. Andrzej Górski, the patient information sheet and informed consent form regarding phage treatment at the PTU, both in Polish and English, facilitate compliance with the guaranteed rights of the patient during therapy and provide patients with all the necessary information. All patients are required to carefully read the entire document and confirm that they understand the purpose, principles and risks associated with phage therapy. The English version of the document, last revised in May 2021, includes the following sections:

- The purpose and basics of experimental phage therapy (including a brief description of the method of producing phage preparations);
- The principles of conducting experimental phage therapy (including admission criteria, ways of administering the formulation and follow-up visit description);
- Possible risks and undesired effects that patients may encounter while undergoing phage treatment;
- Mandatory insurance and protection of patients’ rights;
- The costs of the experimental treatment;
- The right to withdraw from the treatment at any time;
- Description of cases when treatment is terminated by the physician;
- Procedures in the event of patient doubts and the appearance of new information;
- Contact details for the PTU and physician supervising the experiment;
- A two-page informed consent form which also includes consent to the storage and processing of the patient’s personal data (in accordance with the General Data Protection Regulation) to be signed by the patient (or their legal representative) and the physician who provided information on the experimental phage therapy.

Modification of the protocol in June 2008 extended criteria that allowed the PTU physician to evaluate previous antibiotic treatment applied by the patient as ineffective. Such an evaluation could also be based on the opinion of an external medical specialist. This practice soon led to another significant modification in the treatment regimen. Physicians working at the PTU have begun requiring patients interested in phage treatment to have a referral from their general practitioner. Thanks to that change, a general practitioner, being a specialist who is best informed about the patient’s health, is informed about the patient’s plans and is able to raise any concerns related to their medical condition before any procedures have started. This is especially important in oncological patients whose immune system might be weakened due to previous therapies. Usually, physicians at the PTU try to be more insightful than general practitioners or attending oncologists so that all potential issues are resolved at the beginning of the treatment. Notably, such an approach is even more rigorous compared to clinical trials where general practitioners are informed after patient enrollment, and they are only asked for relevant medical information rather than permission. The referral applicable at the PTU requires the general practitioner to provide information about the bacterial infection a given patient is dealing with, documented previous targeted treatment that turned out to be ineffective and their opinion on the patient’s ability to follow the principles of phage treatment conducted on an outpatient basis. It also includes brief information regarding the basics of phage therapy.

In view of the above, it is clear that the availability of phage preparations in Poland is strictly limited only to patients qualified for phage treatment at the PTU. Certainly, such preparations are not marketed across the country, and it is impossible to purchase them in Polish drug stores, with or without a prescription. Contrary to such an approach, wide availability of phage preparations is standard practice in other countries with a history of phage therapy, e.g., Georgia or Russia, where they can be purchased even online [38,39]. Limited access to phage preparations also applies to hospitals that are interested in phage treatment. Each hospital must obtain approval from the competent bioethics committee with the PTU’s help regarding templates of required application forms and guidelines. The number of hospitals, medical centers, outpatient clinics and institutes that implemented phage treatment in their patients using phage preparations from the PTU in the years 2005–2021 exceeded 80 and varied from 14 in the first year of the PTU’s activity to only 1 in 2020 due to the COVID-19 pandemic outbreak and the accompanying crisis in healthcare.

Another milestone in the history of the PTU was the introduction in the treatment regimen of purified staphylococcal phage cocktails that consist of three phages for topical and oral use. Preparations containing phages selected based on research conducted at the Bacteriophage Laboratory and treatment outcomes of patients registered at the PTU were manufactured by IBSS BIOMED S.A., a Polish biotechnological company with facilities and experience suitable for delivering preparations with the expected GMP and GLP standards [1,31]. Although no serious side effects were observed in patients after the administration of phage lysates, the introduction of purified preparations was an important step in allowing new clinical data to be obtained. Our phage lysates intended for oral use undergo quality control based on the European Pharmacopoeia which includes visual testing (impurities visible to the naked eye), and testing of lytic activity, sterility (aerobic and anaerobic bacteria cultures), stability (shelf-life testing) and pH (via potentiometric transduction). In purified phage preparations, additional tests are performed such as bacterial endotoxin testing, protein profiles and testing for the content of stabilizers. The bioethics committee approved the implementation of purified phage preparations in 2013, with the first patients receiving such cocktails a year later. In 2015, the same committee approved phage treatment in minors aged 6 years or older. Another approval from 2016 was associated with intravesical administration of phages, through a catheter into the bladder, in males and females with urinary tract infections. These approvals show steady progress regarding phage treatment at the PTU based on substantive premises and clinical data. Phage therapy conducted at the PTU is registered in the ClinicalTrials.gov database with the identifier NCT00945087.

Patients treated at the PTU have also been monitored for remote phage safety, and our observations suggest a lack of significant side effects or serious complications even after seven years after completion of phage therapy administered locally or orally [40]. The genomic analysis of our staphylococcal phages has shown that they are free of homologs of known virulence factors and determinants of antibiotic resistance. Analysis of the remaining phages is underway while we exclude those with potential harmful effects on patients.

### 4.4. Treatment-Related Activity

**Figure 3:**
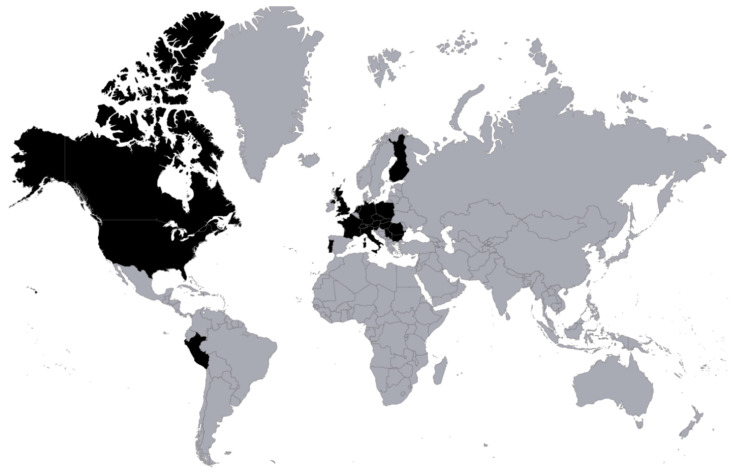
Countries of origin (highlighted in black) of registered patients seeking help at the PTU in the years 2005–2021 (source: internal documentation).

Starting from the first year of its operation, the PTU has been actively registering and consulting patients from Poland and abroad. Although the numbers have never been a goal in themselves and the number of patients was strictly limited for the reason mentioned above (detailed medical history, strict exclusion criteria, costs), the summary of the PTU’s activity comprises hundreds of treated patients, and the treatment outcomes have been summarized numerous times in peer-reviewed articles and books [25,31,35,40,41,42,43,44]. A detailed list summarizing the PTU’s activity in the years 2005–2021 is provided in Table 1, whereas Figure 3 presents countries from which patients sought help at the PTU. Among over 2000 patients registered at the PTU, a plethora of different infections requiring different phage types and routes of administration have been diagnosed. A detailed description is shown in Table 2.

Phage treatment at the PTU has always been supplemented by research activity at the Bacteriophage Laboratory. The main procedure that is mandatory in order to deliver individually selected therapeutic phage preparations to the patient is so-called phage typing [31,45]. Thus far, the laboratory has conducted nearly 3000 phage typing procedures based on samples collected from the patients, of which over 80% resulted in finding active phages against the tested pathogen. These results confirm that constantly expanded and updated phage collection retains its therapeutic potential. For instance, our staphylococcal phages preserve the broadest lytic spectrum, reaching 95%, whereas coliphages show an 86% mean lytic spectrum against *E. coli* strains, including those that are ESBL-positive [45]. A summary of phage typing conducted at the Bacteriophage Laboratory in the years 2006–2021 is presented in Table 3. In the case of repeated phage typing of one strain isolated from the same patient and resulting in the same outcome (sensitivity to the tested phages), such an assay was counted only once. Thus, the actual amount of phage typing performed for the patients registered at the PTU may even be twice as large. The most common bacteria isolated from the patients belong to the *Staphylococcus*, *Enterococcus* and *Escherichia* genera, which corresponds to the number of the most popular therapeutic phage lysates prepared in the laboratory. These numbers are presented in detail in Table 4 and Table 5.

Phage collection used in the treatment of PTU patients is as old as the Hirszfeld Institute and dates back to 1948 when Prof. L. Hirszfeld initiated the isolation of the first phages [45]. Although it served as a primary source of phages for over 50 years, its vast expansion took place after the PTU’s establishment, mainly thanks to numerous research programs funded by the European Union. The first long-term project entitled “*Optimization of the production and characterization of bacteriophage preparation for therapeutic use*” was conducted in the years 2007–2014 with funds from the National Centre for Research and Development under the Innovative Economy Operational Programme [46]. Such projects allowed the expansion of the collection, including the collection of plasmid- and prophage-free bacterial hosts used to produce therapeutic phage lysates [25]. Only between 2014 and 2019 did phage collection at the Hirszfeld Institute expand from 688 to 859 phages, including those lytic against antibiotic-resistant bacteria such as MSSA, MRSA and MRCNS strains of *Staphylococcus*, HLAR, HLGR and VRE strains of *Enterococcus*, ESBL+ strains belonging to *Escherichia*, *Klebsiella*, *Stenotrophomonas*, *Serratia, Proteus* and *Morganella* and even carbapeneme-resistant strains of *Klebsiella pneumoanie* [45]. Since the first year of the PTU’s activity, its phage collection has been enriched with completely new types of phages almost every year. In 2005, *Serratia* phages were added; in 2006, phages against *Stenotrophomonas* were added; in 2007, phages against *Acinetobacter* and *Burkholderia* were added; in 2008, phages against *Citrobacter*, *Morganella* and *Enterococcus faecium* were added (although phages against *E. faecalis* were deposited in our collection before that year); in 2009, phages against *Raoultella*, *Comamonas*, *Aeromonas* and *Enterobacter hormaehei* (previously divided into two species, *E. cloacae* and *E. diversus*) were added; and in 2011, phages against *Streptococcus* were added.

In conclusion, a great number of patients qualified for treatment at the PTU over the last 15 years, and research involving nearly 900 phages deposited in our collection has allowed unique and important findings to be obtained at a global level regarding clinical tolerability, immunogenicity and treatment of antibiotic-resistant infections as well as providing an in-depth analysis of clinical results through extensive observational studies [25].

### 4.5. Research Activity

Publishing activity has always been the key element of the Bacteriophage Laboratory and one of the main activities of the PTU. For 15 years since the PTU’s initiation, our team has published over 100 peer-reviewed articles in English, both review and original articles. Listing all of them is unnecessary as the vast majority of them are indexed in the Medline database and are widely available through an open access license at the following website: https://pubmed.ncbi.nlm.nih.gov. By entering the names of the most popular authors (Górski A., Międzybrodzki R., Dąbrowska K., Weber-Dąbrowska B.) in the search window, all articles will appear in the search results of the above-mentioned database. It is worth mentioning that our catalog includes pioneering, widely cited publications on phage therapy in humans, phage immunogenicity and phage molecular biology. This catalog is not limited to articles. The most significant positions include two books edited by physicians from the PTU. The first one entitled “*Phage Therapy: Current Research and Applications*” was issued in 2014 by Caster Academic Press and edited by Jan Borysowski, M.D., Ph.D., Assoc. Prof. Ryszard Miedzybrodzki, M.D., Ph.D. and Prof. Andrzej Gόrski, M.D., Ph.D. [47]. In 2019, the second book entitled “*Phage Therapy: A Practical Approach*” was released by Springer and includes the same editors as listed above [48]. Both books received favorable reviews. In 2015, the journal *Clinical Infectious Diseases* published a review of the first book by Keen E.C. and Adhya S.L. [49], in which the book was described as a valuable resource of phage-related knowledge. Another review of the same position published in *The Lancet Infectious Diseases* [50] praised the authors for shedding light on how phages could become a powerful weapon against a growing threat from antibiotic-resistant bugs. In May 2020, Prof. Stephen Abedon in his review published by *The Lancet Infectious Diseases* stated that the latter volume addresses, for the first time, legal issues related to the implementation of phage therapy at a clinic [51]. Both positions as well as the most important articles on phage therapy have been described in our previous paper summarizing the history of phage therapy in Poland [1]. The popularity of our works is reflected in the number of citations, which in the case of some authors reached, according to the Scopus and Web of Science databases, a few thousand, while several hundred citations are common even among Ph.D. candidates (Table 6).

Since 2005, in addition to the published work, our team has submitted 20 international patent applications covering methods of phage application, purification and production and antibacterial properties of phage-derived lysins. Moreover, the daily activity of the PTU and the Bacteriophage Laboratory has been greatly supported by funds derived from a plethora of different financing institutions, including, but not limited to, the National Centre for Research and Development, the Polish Agency for Enterprise Development and the National Science Centre. Phage scientists and physicians at the PTU and both phage labs were involved in the implementation of 33 phage-related projects for a total amount of PLN 36,000,000 (ca. USD 8,500,000). The list of the most funded phage-related projects with an allocation of over PLN 500,000 (ca. USD 120,000 as of April 2022) is presented in Table 7.

### 4.6. Polish and International Recognition

Shortly after its establishment, the PTU became a role model both in Poland and abroad for people interested in implementing phage treatment in their countries. As mentioned above, Gill and Young proposed treating patients in the form of compassionate use already implemented at the Polish PTU which would potentially speed up and facilitate phage therapy across the USA [22]. The first years of the PTU’s activity were noticed by the medical and scientific community, which was reflected in articles and recognitions granted to the team. In 2006, the achievements of the Polish group were the subject of a report in a leading scientific periodical, *Nature Medicine* [52]. In an article entitled “Bug Killers”, the author emphasized that, despite the lack of randomized, placebo-controlled studies, Polish research was well documented. A year later, a Polish mainstream newspaper, *Gazeta Wyborcza*, published an article on an e-petition posted on the website of the British government in which the Brits demanded action from their authorities to enable treatment with phages from Poland and possibly implement similar treatment in the UK [53]. Although the efforts failed, it was one of the first signals that the activity of the Polish PTU was being noticed outside of Poland. In the same year, Prof. Andrzej Górski received recognition from the Polish edition of *National Geographic Traveler* “*Travelers 2007*” in the category “Scientific discovery of the year—Unique experimental phage therapy” [54]. Notably, the choice was partly made by the readers, which was another sign of the growing popularity of this form of treatment in Poland thanks to the PTU’s activity. On the wave of this popularity, the year 2009 brought another team recognition, the “Golden Scalpel 2008” for “Innovation in Polish medicine”, awarded by the editorial office of *Puls Medycyny*, the largest independent newspaper and opinion-forming magazine in Poland intended for the medical community, physicians and managers of the medical sector [55]. It should be noted that all of these aforementioned achievements and hype took place when the Western world was still in its pre-phage era with little interest among scientists and corresponding outcomes [56].

In the following years, interest in the Wrocław center did not diminish, which was also associated with the growing popularity of phage treatment globally. In 2017, a video production service, Renegade Pictures, produced a one-hour TV report on phage therapy entitled Michael Mosley vs. The Superbugs, which premiered in the UK (BBC Four) on 17 May 2017 [57]. The author described in it the activity of the Wrocław center and key aspects of phage treatment conducted at the PTU. A year later, German TV station ZDF released a documentary directed by Torsten Mehltretter entitled “*Heilen ohne antibiotika”* (*Treating Without Antibiotics*) describing phage centers in the so-called “Eastern Bloc”, including a summary of the PTU [58]. On 5 February 2019, AVROTROS, a Dutch radio and television broadcaster that is part of the Dutch public broadcasting system, released a detailed story of one of the PTU’s patients entitled “*Bacteriophagen*” (*Bacteriophages*) as part of a series called “*Dokters van morgen*” (*Tomorrow’s Doctors*) directed by Antoinette Hertsenberg [59]. In the same year, an article published online by EverethNews.pl presented the history of a 19-year-old patient from Peru who sought help at the PTU [60]. In the section describing international recognition, it must be mentioned that in 2019, a new genus of bacteriophages was named after Beata Weber-Dąbrowska, Ph.D. (*Webervirus*) by a decision of the International Committee on Taxonomy of Viruses [1].

**Figure 4:**
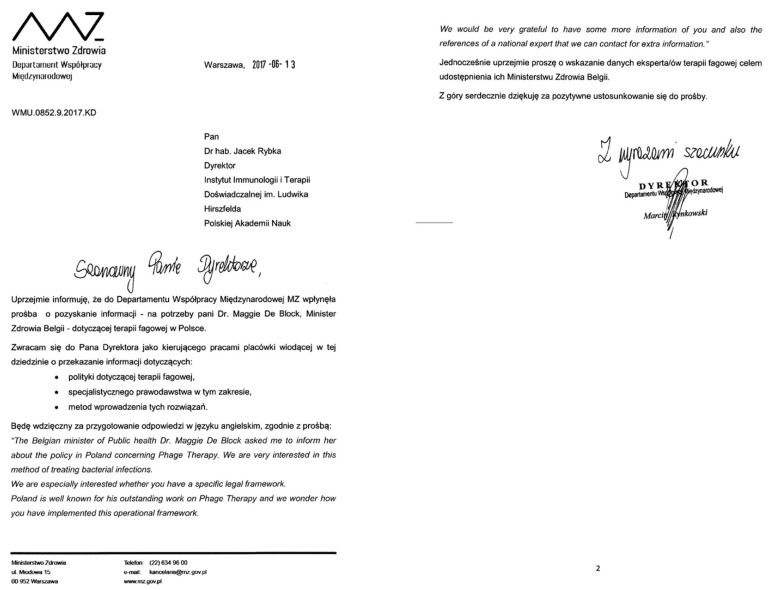
A letter dated 13 June 2017 addressed to the Hirszfeld Institute director forwarding a request from the Belgian Minister of Public Health regarding phage therapy in Poland (in Polish/English).

Phage treatment conducted at the PTU was also met with interest from the state authorities. In 2017, the Belgian Minister of Public Health, Dr. Maggie De Block, requested that the Polish Ministry of Health provide policies and details concerning legal frameworks that are in force in Poland, describing our country as “well known for his outstanding work on Phage Therapy” (Figure 4).

In December 2021, the high position of the PTU was confirmed in several letters of recommendation addressed to Prof. Andrzej Górski, M.D., Ph.D. and authored by prominent phage scientists such as Prof. Andrew M. Kropinski, Ph.D. from the University of Guelph, Jerzy W. Kupiec-Wegliński, M.D., Ph.D. from the University of California (Los Angeles), Michael Skurnik, Ph.D. from the University of Helsinki, Stephen T. Abedon, Ph.D. from Ohio State University and Dr Alain Dublanchet from Villeneuve-Saint-Georges Hospital Center. In addition, Prof. Andrzej Górski, M.D., Ph.D. has been invited to many symposia, meetings, conferences and workshops held by institutions including, but not limited to, the National Institute of Health, the MD Anderson Cancer Institute (USA), the European Medicines Agency and the National Institute of Virology in Beijing.

## 5. Discussion

Certainly, it is not possible to cover all aspects of the PTU’s activity in Wrocław, Poland, in one article. A detailed description of scientific publications is beyond the scope of this article. However, some highlights cannot be omitted to honor the hard and important work of the research team. Scientists and physicians from the PTU and the Bacteriophage Laboratory have given some important, often pioneering insights into the safety and immunogenicity of therapeutic phage preparations [25,31,32,33,34,35,36,37] and hypotheses concerning the anti-inflammatory and immunomodulatory nature of phage interactions, which opens the way to their innovative use in non-bacterial diseases, including COVID-19, in the form of so-called drug repurposing [61,62]. Research conducted by our team in recent years has shown that phages can alter the immune system’s activity, which can be exploited in the treatment of autoimmune diseases, combating transplant rejection and other complications [63,64]. Moreover, we have pointed out that the profile of immune reactions to phages may be phage-specific. Although there are only very limited data on the immunomodulatory effects of phages during clinical phage therapy, some of our assumptions have been confirmed by other authors. For example, Petrovic Fabijan et al. [65] showed that the therapy induced an anti-inflammatory response, and that inflammatory markers decreased in the majority of treated patients. Moreover, a decrease in IL-6 serum levels has been described during phage treatment [66]. Furthermore, recent experimental findings provide ample evidence in support of our hypothesis pointing to the anti-inflammatory action of some phages which may be unrelated to their antibacterial activity. Thus, it has been shown that temperate filamentous phages may exert an immunosuppressive action; interestingly, lytic phages do not have such effects [67]. Recently, a group from Harvard Medical School showed that phages inhabiting the normal human colon elicit an immunomodulatory program in macrophages and intestinal epithelial cells promoting the production of anti-inflammatory cytokines, maintaining hemostasis and protecting against induced colitis in vivo [68]. Bao et al. [69] demonstrated that pretreatment with lytic and temperate phages markedly reduced intestinal inflammation and increased the levels of anti-inflammatory but not pro-inflammatory cytokines. Finally, M13 phage used as a matrix for macrophage cultures induced an anti-inflammatory phenotype in those cells. Thus, phages can polarize macrophages towards the M2 phenotype which suggests that some phage biomaterials could be used for local immunotherapy of inflammatory diseases [70]. Therefore, in the future, a specific phage could be selected for phage therapy taking into account both antibacterial activity and the type of immune response it may evoke, paving the way for the use of specific phages in immunomodulation [71]. Notably, recent data suggest that phages modulate cognitive function, so it may well be that phage therapy could enhance performance in executive processes and verbal memory [72].

Scientific activity developed in parallel with medical activity at the Hirszfeld Institute has played a significant role in building the foundation for the development of phage therapy globally. Current estimates indicate that by 2050, ten million people will die from antimicrobial resistance (AMR), i.e., more than from cancer. AMR is considered one of the greatest threats to humanity while the introduction of new antibiotics is very limited. Notably, we do not have to wait until 2050. A global survey showed that in 2019, antimicrobial resistance already killed more people than HIV/AIDS or malaria [73]. Thus, the phage therapy program implemented at the PTU, the first such center in the European Union, creates an opportunity to solve one of the main problems in healthcare. The further development of phage therapy may contribute to the progress of medicine and a significant improvement in the life and functioning of our civilization. A measurable social benefit is the enrichment of healthcare with a new, very promising therapeutic method.

The current setup of our phage therapy center responds to the actual needs of patients reporting to us while we work on a non-profile basis (in fact, every year, the center has been running economical deficits as the costs charged do not match the actual production costs and very moderate wages paid to the personnel (more data are available on our website)). Since other phage therapy centers are being opened, the number of patients coming to us for treatment is decreasing. It could be expected that the successful clinical trials and resulting eventual introduction of phage therapy as an accepted standard treatment would lead to further transformation of the role of our center. Thus, we have been considering initiating clinical studies on the immunomodulatory and anti-inflammatory potential of phages unrelated to their antibacterial action in disorders of immunity. We hope that our unit could play a pioneering role in this challenging and promising project, opening a new era of repurposed phage therapy.

As the PTU continues its activity, despite several obstacles such as the recent COVID-19 pandemic outbreak that significantly reduced the number of registered patients, we continue our efforts to deliver the most reliable and proven scientific evidence for phage therapy. Such attempts do not distract us from the belief that only clinical trials will provide the final answer on the efficacy and safety of phage therapy conducted at the PTU and subsequently opened phage centers around the globe.

## Figures and Tables

**Table 1:** The PTU’s activity in the years 2005–2021 (source: internal documentation).

Category	Numbers
Patients registered at the PTU	**2207** (including **126** in Kraków branch and **13** in Częstochowa branch)
Visits/consultations registered at the PTU	**6207** (including **303** in Kraków branch and **16** in Częstochowa branch)
Patients qualified for experimental phage therapy at the PTU	**791** (including **37** in Kraków branch and **0** in Częstochowa branch)

**Table 2:** Type of infection in patients registered at the PTU in the years 2006–2021 * (source: internal documentation).

	Type of Infection										
Year	UTI	GTI	PI	URTI	LRTI	P	SI	C ^	O	BNI	BLI
2006	25	7	61	30	6	12	10	-	3	4	2
2007	9	2	27	3	5	11	-	-	2	3	-
2008	19	6	55	22	-	16	7	1	3	5	-
2009	21	7	5	17	-	16	57	-	3	3	1
2010	15	9	7	21	-	6	42	1	4	6	2
2011	13	10	11	25	-	14	27	-	3	14	2
2012	19	5	19	20	2	13	37	1	3	12	-
2013	9	2	7	13	1	15	29	-	2	9	-
2014	19	9	10	16	3	11	41	-	3	7	1
2015	31	10	15	22	-	16	41	-	3	21	2
2016	41	8	10	24	-	22	48	-	6	13	1
2017	31	11	2	20	-	15	36	2	2	10	-
2018	38	5	5	20	-	21	36	-	6	10	1
2019	57	11	11	23	-	18	40	-	3	12	-
2020	25	3	1	7	-	18	21	-		9	-
2021	47	3	5	13	-	21	27	-	3	11	-
**Total**	**419**	**108**	**251**	**296**	**17**	**245**	**499**	**5**	**49**	**149**	**12**

UTI—urinary tract infections; GTI—genital tract infections; PI—postoperative infections; URTI—upper respiratory tract infections; LRTI—lower respiratory tract infections; P—prostatitis; SI—skin infections; C—conjunctivitis; O—otitis; BNI—bone and joint infections; BLI—blood infections. * The percentage of patients typed for phage treatment in relation to registered patients was approximately 36%. ^ Although the PTU has not been granted authorization to treat patients via the ocular route, some of them received phage preparations intended for oral use.

**Table 3:** Results of phage typing performed for PTU patients in the years 2006–2021 (source: internal documentation).

Phage Typing			
Year	Positive Results (%)	Negative Results (%)	Total Number of Typed Isolates
2006	203 (79.0)	54 (21.0)	257
2007	216 (82.4)	46 (17.6)	262
2008	196 (78.4)	54 (21.6)	250
2009	188 (82.0)	41 (18.0)	229
2010	162 (92.0)	14 (8.0)	176
2011	130 (80.8)	31 (19.2)	161
2012	146 (84.3)	27 (15.7)	173
2013	123 (85.4)	21 (14.6)	144
2014	164 (81.6)	37 (18.4)	201
2015	165 (84.7)	30 (15.3)	195
2016	209 (82.0)	46 (18.0)	255
2017	134 (77.4)	39 (22.6)	173
2018	130 (79.2)	34 (20.8)	164
2019	120 (76.4)	37 23.6)	157
2020	78 (79.6)	20 (20.4)	98
2021	79 (85.0)	14 (15.0)	93
**Total**	**2443 (81.8)**	**545 (18.2)**	**2988**

**Table 4:** The most abundant bacterial strains isolated from PTU patients in the years 2006–2021 (source: internal documentation).

	Bacterial Strain										
Year	*S. aureus*	*E. faecalis*	*E. coli*	*Kl. pneum.*	*Ent. cloac.*	*P. mirabilis*	*P. aeruginosa*	*S. marcescens*	*A. baumannii*	*M. morganii*	Total
2006	129	37	34	6	2	5	24	2	3	1	**243**
2007	111	37	35	1	1	5	44	3	2	2	**241**
2008	103	38	42	1	4	5	31	2	2	-	**228**
2009	115	30	31	-	3	2	31	-	1	-	**219**
2010	30	24	18	8	2	2	35	2	3	3	**127**
2011	69	22	19	6	1	3	36	2	2	2	**162**
2012	89	14	27	7	-	3	36	1	2	2	**181**
2013	69	17	12	3	3	4	44	1	1	-	**154**
2014	81	29	23	11	5	3	45	-	1	-	**198**
2015	66	26	38	19	3	6	28	-	1	-	**187**
2016	86	27	38	34	6	5	44	2	1	3	**246**
2017	58	24	43	30	3	5	30	2	7	2	**204**
2018	55	32	39	26	4	4	32	5	2	1	**200**
2019	42	19	36	34	4	4	43	4	-	2	**188**
2020	25	19	24	17	3	4	6	-	-	2	**100**
2021	24	18	31	20	3	5	18	1	1	1	**122**
**Total**	**1152**	**413**	**490**	**223**	**47**	**72**	**527**	**26**	**29**	**21**	**3000**

**Table 5:** Type of the most popular phage preparations delivered to the patients treated at the PTU in the years 2006–2021 (source: internal documentation).

	Type of Phage Preparation					
Year	*Staphylococcus*	*Enterococcus*	*Escherichia*	*Klebsiella*	*Pseudomonas*	Total
2006	105	35	26	0	4	**170**
2007	102	30	17	0	15	**164**
2008	98	21	17	0	10	**146**
2009	120	21	16	2	21	**180**
2010	67	35	18	9	20	**149**
2011	46	28	19	4	11	**108**
2012	73	9	43	11	22	**158**
2013	80	24	18	3	45	**170**
2014	84	33	30	6	39	**192**
2015	58	27	59	12	15	**171**
2016	84	33	34	14	20	**185**
2017	59	18	32	9	18	**136**
2018	66	21	23	19	22	**151**
2019	50	27	27	18	27	**149**
2020	24	20	10	9	1	**64**
2021	27	18	32	12	16	**105**
**Total**	**1143**	**400**	**421**	**128**	**306**	2398 *

* One pack contains 33 vials of 10 mL each.

**Table 6:** Scientific metrics of the current employees of phage-related units of the Hirszfeld Institute with the greatest number of citations * according to the Scopus and Web of Science databases (as of 13 April 2022).

Employee	Citations and H-Index acc. to Scopus	Citations and H-Index acc. to Web of Science
Prof. Andrzej Górski, M.D., Ph.D.	5698 (44)	5405 (43)
Beata Weber-Dąbrowska, Ph.D.	3691 (40)	2752 (33)
Assoc. Prof. Ryszard Międzybrodzki, M.D., Ph.D.	2522 (27)	2302 (26)
Prof. Krystyna Dąbrowska, Ph.D.	2072 (27)	2218 (30)
Wojciech Fortuna, M.D., Ph.D.	1517 (19)	1396 (17)
Marzanna Łusiak-Szelachowska, Ph.D.	1026 (17)	964 (17)
Ewa Jończyk-Matysiak, Ph.D.	993 (19)	965 (19)
Assoc. Prof. Sławomir Letkiewicz, M.D., Ph.D.	937 (12)	923 (11)
Paweł Rogóż, M.D.	670 (8)	660 (8)
Barbara Owczarek, M.Sc., Eng.	569 (13)	514 (13)
Maciej Żaczek, M.Sc.	545 (12)	505 (11)
Paulina Miernikiewicz, Ph.D.	542 (12)	522 (12)
Zuzanna Kaźmierczak, Ph.D.	475 (9)	457 (9)

* Autocitations excluded.

**Table 7:** Chronological list of the most funded phage projects conducted at phage-related units of the Hirszfeld Institute since 2005.

Project Title	Principal Investigator	Funds (PLN) *	Funding Institution	Years
Optimization of the production and characterization of bacteriophage preparation for therapeutic use	Prof. Andrzej Górski, M.D., Ph.D.	6,026,360	National Centre for Research and Development	2009–2014
Use of bacteriophages to develop antibacterial preparations in veterinary	Beata Weber-Dąbrowska, Ph.D.	3,170,000	Polish Agency for Enterprise Development	2013–2015
Biological stabilization of drinking water	Beata Weber-Dąbrowska, Ph.D.	2,200,000	National Centre for Research and Development	2013–2015
Identification of the influence of bacteriophages with therapeutic potential on mammalian cellular functions and the immune system	Prof. Krystyna Dąbrowska, Ph.D.	1,445,000	National Science Centre	2013–2017
Innovative bacteriophage preparation for the treatment of diabetic foot	Beata Weber-Dąbrowska, Ph.D.	6,188,704	National Centre for Research and Development	2013–2015
Antiviral activity of bacteriophages	Prof. Andrzej Górski, M.D., Ph.D.	929,600	National Science Centre	2014–2017
The differentiation of the immunological reactivity of endolysins as a factor determining the differences in their antimicrobial efficacy in vivo	Prof. Krystyna Dąbrowska, Ph.D.	1,108,800	National Science Centre	2016–2019
Development and implementation of a bacteriophage preparation used in the treatment and prevention of American foulbrood	Ewa Jończyk-Matysiak, Ph.D.	4,524,769	National Centre for Research and Development	2017–2019
Gastric microbiome in people infected with Helicobacter pylori	Prof. Krystyna Dąbrowska, Ph.D.	1,751,400	National Science Centre	2019–2022
PhageScan: Identification of bacteriophage epitopes significant to human health	Prof. Krystyna Dąbrowska, Ph.D.	2,361,600	National Science Centre	2020–2024
Study of the composition of a bacteriophage preparation specific to multidrug-resistant Acinetobacter baumannii clinical strains	Natalia Bagińska, M.Sc. Eng.	800,000	National Centre for Research and Development	2021–2023
Mechanisms of phage-derived “dark matter” interactions with the immune system of mammals	Prof. Krystyna Dąbrowska, Ph.D.	2,038,200	National Science Centre	2021–2024

* PLN—Polish zloty (USD 1 = PLN 4.27 as of 8 April 2022; source: National Bank of Poland).
